# The effects of aerobic exercise and heat stress on the unbound fraction of caffeine

**DOI:** 10.3389/fphys.2024.1370586

**Published:** 2025-01-06

**Authors:** Mackenzie McLaughlin, Kaye Dizon, Ira Jacobs

**Affiliations:** ^1^ Human Physiology Research Unit, Faculty of Kinesiology and Physical Education, University of Toronto, Toronto, ON, Canada; ^2^ Department of Clinical Research, Cleveland Clinic Canada, Toronto, ON, Canada; ^3^ Department of Pharmacology and Toxicology, Faculty of Medicine, University of Toronto, Toronto, ON, Canada; ^4^ The Tannenbaum Institute for Science in Sport, University of Toronto, Toronto, ON, Canada

**Keywords:** exercise pharmacokinetics, plasma protein binding, metabolic ratio, ultrafiltration, Centrifree^®^, exercise hemodynamics, Q10 effect

## Abstract

**Introduction:**

The fraction of drug circulating in the blood that is not bound to plasma proteins (*f*_*u*_) is considered pharmacologically active since it readily binds to its receptor. *In vitro* evidence suggests that changes in temperature and pH affect the affinity of drug binding to plasma proteins, resulting in changes in *f*_*u*_. In light of the well-established effects of exercise on body temperature and blood pH, we investigated whether an increase in blood temperature and decrease in pH facilitated through passive heating and exercise translated to a change in the *f*_*u*_ of caffeine.

**Methods:**

Ten healthy participants (4 females and 6 males; age: 21.9 ± 2.7 years [means ± SD]) ingested 3 mg/kg of anhydrous caffeine on two separate occasions comprised of a control trial involving 105 min of rest, and an experimental trial involving 10 min of passive heating, followed by 20 min of cycling at 55% $\dot{V}O_{2\text{peak}}$ , and then 10 sprint intervals at 90% $\dot{V}O_{2\text{peak}}$ . Venous blood was sampled and the plasma was processed via ultrafiltration to quantify the *f*_*u*_ of caffeine and its major metabolite, paraxanthine.

**Results:**

The exercise protocol resulted in maximal increases in core temperature of 1.37°C ± 0.27°C and lactate of 10.34 ± 3.33 mmol/L, and a decrease in blood pH of 0.12 ± 0.051 (all *p* < 0.05), which did not affect the *f*_*u*_ of caffeine (baseline: 0.86 vs post-exercise: 0.75; *p* = 0.30) or paraxanthine (baseline: 0.59 vs. post-exercise: 0.70; *p* = 0.11). Furthermore, the rate of metabolism of caffeine assessed through the metabolic ratio ([paraxanthine]/[caffeine]) did not differ between resting and exercise trials.

**Discussion:**

Therefore, the changes in blood temperature and pH in this study did not affect the *f*_*u*_ of caffeine or paraxanthine.

## 1 Introduction

Evaluating potential interactions when medications are ingested by individuals increasing their physical activity is becoming more important given the rise in drug prescriptions (OECD, 2019) and exercise recommendations (Task Force on Community Preventive, 2002). Previous drug-exercise investigations have focussed on exercise-induced hemodynamic changes on drug pharmacokinetics (PK) (Sweeney, 1981), yet drug binding in blood remains unstudied. This is puzzling since exercise is associated with physicochemical and biochemical changes in the blood, with temperature and acidity being major factors that both affect drug binding and, thus, its efficacy. Therefore, a patient given prescriptions for both exercise and medications may experience unintended drug effects. Examining exercise’s effect on drug-plasma protein binding will add to our repository of drug interactions for personalized medicine and provide insight into exercise as a modality to elicit specific physiological changes that can predictably change a medication’s effect.

Drugs travel in the blood in two forms—that which is bound to plasma proteins, known as the bound fraction (*f*_*b*_ *),* and that which is not bound to plasma proteins, known as the free/unbound fraction (*f*_*u*_). The degree and relative affinity of drug binding to plasma proteins (e.g., albumin, α1-acid glycoprotein [AGP], and lipoprotein) is based on the drug’s physicochemical properties, with basic drugs binding well to AGP, and acidic and neutral drugs preferentially binding to albumin (Brown et al., 2010). Since the *f*_*b*_ is bound to proteins, it is not available to bind to its receptor and is, thus, rendered inactive. In contrast, the *f*_*u*_ is free in the circulation and can: 1. bind to its receptor to elicit a pharmacological response; 2. move to extravascular tissues, increasing the drug’s volume of distribution (V_d_); and 3. interact with enzymes and transporters, increasing the rate of drug metabolism and excretion (Roberts et al., 2013). For example, a 9.7% increase in the %*f*_*u*_ of the antibiotic, ertapenem, resulted in a 112% increase in its clearance (CL) and 160% increase in its V_d_ (Burkhardt et al., 2007). Thus, drug binding is highly relevant to understanding both drug PK as well as pharmacodynamics (PD).

All drugs have an inherent affinity for plasma proteins which, in exception to pregnancy and aging, remains relatively constant for healthy individuals under normal physiological conditions (Kremer et al., 1988). However, disruption of the drug-protein complex through competitive binding with exogenous substances and conformational changes from physiological perturbations can alter the degree of drug-protein binding. Competitive binding is commonly observed with drug-drug interactions, whereby drug A will competitively bind to a similar binding spot occupied by drug B, effectively disrupting its interaction and increasing drug B’s *f*_*u*_. For example, valproic acid displaces phenytoin from a shared albumin binding spot because of its higher association constant (McElnay and D’Arcy, 1983). Conformational changes to proteins from endogenously-generated perturbations can affect ligand-binding sites, thus, affecting drug-protein affinity. For example, *in vitro* studies give evidence that changes in blood pH and temperature can affect a drug’s *f*_*u*_ *.* In pooled human serum spiked with propranolol (a beta blocker with %*f*_*u*_ < 10%), a decrease in pH from ∼7.6 to ∼6.6 was associated with an increase in %*f*_*u*_ from 6% to 10.5% (Paxton and Calder, 1983). The same study showed that an increase in temperature from 25°C to 30°C was associated with a ∼1.5% increase in propranolol’s %*f*_*u*_ (Paxton and Calder, 1983). This effect is rather common. A review of *in vitro* studies investigating >20 compounds revealed that all of the basic drugs investigated experience an increase (e.g., ranging from 3.5% [practolol] to 136% [fentanyl]) in *f*_*u*_ when pH is decreased, while acidic drugs exhibit unpredictable changes in *f*_*u*_ (e.g., ranging from −15% [etopside] to 95% [tenoxicam]) (Hinderling and Hartmann, 2005). The therapeutic significance of alterations in *fu* of these magnitude are maximized in drugs that: 1. are highly protein bound, 2. have a high intrinsic clearance (CL_int_); or 3. are not titrated to the pharmacological response (Roberts et al., 2013). The first point is exemplified with (R)-warfarin, a commonly prescribed oral anticoagulant that has a plasma protein binding of 99%, which, if decreased to 97%, for example, would result in a 2-fold increase in *f*_*u*_, which would affect V_d_ and CL.

Mechanistically, increasing the temperature results in increased enzyme activity, known as the Q_10_ effect, up until a threshold where the enzyme’s tertiary (i.e., globular) and secondary structures are denatured (Sinha et al., 2008). Changes in pH will alter the degree of ionization and the resulting charge on amino acid side chains (Baler et al., 2014). In the case of albumin, deviating the pH from 7.4 decreases α-helical and increases β-sheet structures, with alkalinic conditions (e.g., pH = 9) reducing overall charge and promoting the N-B (normal-to-base) transition and acidic conditions resulting in the N-F (normal-to-fast) transition at pH = 4 (Peters and Peters, 1995). These conformational changes are reversible up to the extremes of pH < ∼2 and > ∼9, beyond which disulfide bonds are cleaved and, in basic conditions, tyrosyl hydroxyl groups are deprotonated (Peters and Peters, 1995). These changes can be further accelerated by fluctuations in calcium content, urea, and temperature (Peters and Peters, 1995), which are all increased in some capacity during exercise (Foran et al., 2003), and, cumulatively, may promote the conformational changes to albumin within the physiological pH range. For example, the N-B transition at higher pH values is augmented with increasing calcium concentration, which, in the case of warfarin, led to higher binding with albumin (Wilting et al., 1980).

The normal blood pH range is 7.38–7.44, yet exercise above the anaerobic threshold results in the formation and immediate dissociation of lactate to its ion (La^−^) and H^+^, which can decrease blood pH to 6.8 during exhaustive exercise (McArdle et al., 2007). Exercise also increases metabolic heat production, elevating core body temperature (T_c_), with values of >41°C being reported in athletes during competition (Byrne et al., 2006). Given that *in vitro* experiments have characterized changes in drug *f*_*u*_ resulting from changes in the pH and temperature of a drug’s medium, and exercise is associated with changes in blood pH and increased temperature, it is reasonable to speculate that exercise has the potential to affect a drug’s *f*_*u*_. The current investigation is the first to assess in humans the effects of acute exercise on a drug’s *f*_*u*_. Because of the potential risk for aberrant drug effects, we chose to study these interactions using caffeine, the world’s most consumed drug that is toxic at doses ∼30-fold higher than the average daily intake (Nawrot et al., 2003). Moreover, any change in its %*fu* is likely to be reflected in its efficacy, which will be of interest to the ∼75% of professional athletes who ingest caffeine as an ergogenic aid (Aguilar-Navarro et al., 2019). An increase in the %*fu* may cause a stronger ergogenic effect, which may engender recommendations for a lower dose while a decreased %*fu* may engender the opposite.

Caffeine is a weak base that has been shown to bind to albumin (Wu et al., 2009); yet, information about its binding to AGP is scant. After being absorbed within 45 min, caffeine distributes throughout well-hydrated tissues with a V_d_ of 0.5–0.8 L/kg and is primarily (90%) metabolized by the enzyme, CYP1A2, to paraxanthine (84% of CYP1A2 metabolites), which makes caffeine a commonly used probe drug to phenotype 1A2 activity (Nehlig, 2018). Caffeine experiences relatively low protein binding (%*f*_*u*_ = 70–90%) (USIM, 2001) and few studies have evaluated changes in its binding capacity. Comparison of caffeine *f*_*u*_ between young (25–30 years) and elderly (66–78 years) individuals using CF-25 ultrafiltration ‘cones’ revealed that age is associated with a decrease in plasma albumin concentration (45.9–41.13 g/L) but is not associated with a change in %*f*_*u*_ (Blanchard, 1982a). Another study using fluorescence spectroscopy to evaluate albumin-caffeine interactions found a decrease in the apparent binding constant (*K*_*b*_) and number of binding sites with increasing temperatures, giving evidence that the albumin-caffeine complex may dissociate at higher temperatures and increase the *f*_*u*_ of caffeine (Wu et al., 2009). Therefore, the purpose of the current study was to clarify if the *f*_*u*_ of caffeine is influenced by exercise-mediated changes in body temperature and blood pH changes in healthy humans. The study was designed to test the hypothesis that increases in body temperature due to passive heating would increase *f*_*u*_ *,* and that high intensity exercise—but not low intensity exercise—would further increase *f*_*u*_ via decreases in blood pH.

## 2 Methods

### 2.1 Participants

A sample size of 10 was selected based on calculations provided by Lammers et al. (2018) who suggest—based on a *post hoc* power analysis of their values—a sample size of 9 participants will have a power ≥98% in detecting a 15% difference with significance at α < 0.05 in free fractions of caffeine between control and fasting conditions. Since the current study used similar statistical parameters to calculate sample size (with the exception of a more liberal power of >80%) and considering potential participant attrition and methodological/technical challenges that may result in missing data, we recruited a total of 10 participants (4 females) who were briefed on the study details, completed pre-screening questionnaires, including the CSEP Get Active Questionnaire (CSEP, 2017), and provided written informed consent. This study was approved by the University of Toronto Health Sciences Research Ethics Board (approval # 37008).

### 2.2 Study design

The study followed a repeated-measures, counterbalanced, cross-over study design that had participants visit the lab on three occasions. The first was to perform baseline testing while the two subsequent visits consisted of resting (Rest), and combined heat-and-exercise (EXS) trials in a random order separated by a minimum of 1 week. The laboratory was set to an ambient temperature of 21–23°C.

### 2.3 Baseline measurements

In addition to providing basic anthropometric measurements, participants performed sex-specific Åstrand cycle ergometry protocols (Heyward and Gibson, 2014) for determination of peak aerobic power ( $\dot{V}O_{2\text{peak}}$ ), with females starting at 50 W and increasing by 25 W every 2 min, and males starting at 100 W and increasing by 50 W every 2 min, until volitional fatigue. Oxygen consumption rates were recorded throughout each test to calculate cycling economy via power- $\dot{V}O_{2}$ linear regression, which was used to calculate intensities that would elicit steady state oxygen uptake equivalents of 55% and 90% of each participant’s $\dot{V}O_{2\text{peak}}$ for the subsequent EXS trials. These intensities were confirmed after a 10 min rest following the test by having participants engage in cycling for 3 min while monitoring $\dot{V}O_{2\text{peak}}$ .

### 2.4 Heat and exercise trial

**FIGURE 1:**
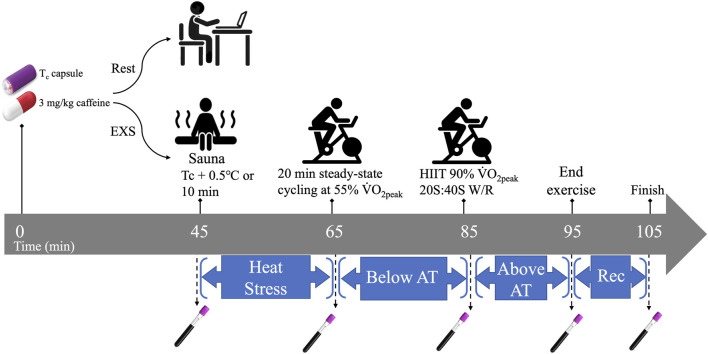
Temporal overview of Rest and EXS trials. AT, anaerobic threshold; HIIT, high intensity intervals; Rec. recovery; T_c_, core temperature; $\dot{V}O_{2\text{peak}}$ , peak aerobic power; W/R, work-to-rest intensity ratio.

An overview of the exercise trial is presented in Figure 1. Participants abstained from caffeine for 48 h before each trial and arrived at the laboratory after a 3-h fast. The trial started with participants ingesting 3 mg/kg of anhydrous caffeine to the closest 25 mg in tablets (Wake Ups, Toronto, Canada)—equivalent in caffeine to a strong cup of coffee—and a Vitalsense® core temperature capsule (Philips Respironics, Pennsylvania, United States) with 1 cup of water 45 min before the start of the trial. Ingestion of caffeine with the core temperature capsule coincided with the timing of maximum drug concentration (T_max_) of caffeine (45 min post-ingestion), and allowed us to minimize participant time requirements and standardize ingestion of the core temperature capsules pre-intervention. This timing between ingestion and T_c_ measurement is clearly shorter than the general recommendation of 3–5 h (Goodman et al., 2009); however, previous experiments in our lab had shown that the core temperature capsule stabilized within 30 min and previous reports show that the capsule would likely have been situated in the small intestine (O'Grady et al., 2020).

The exercise trial began with collection of the first blood sample (t = 45 min post-caffeine ingestion) via venipuncture before passive heating in a sauna until T_c_ increased by 0.5°C or 10 min had elapsed. The participants’ T_c_ was monitored real-time on a laptop within Bluetooth range of the sauna using the companion eqView Professional software (Version 3, Equivital, New York, United States). Participants then returned to the lab and a flexible venous catheter (Nexiva, BD, Franklin Lakes, United States) was inserted in a superficial vein in the antecubital fossa for serial blood sampling and kept patent with saline infusions. Each participant was instrumented with a portable oxygen uptake system (Metamax 3B R1, CORTEX Biophysik Gmbh, Leipzig, Germany), and a heart rate monitor (Polar Electro Oy, Kempele, Finland). Participants then engaged in 20 min of steady-state cycling at 55% $\dot{V}O_{2\text{peak}}$ , which was selected in order to maintain or slightly increase T_c_ without affecting blood pH. Immediately after, participants performed 10 bouts of high-intensity intervals (HIIT) cycling at intensities consistent with 90% $\dot{V}O_{2\text{peak}}$ at work-to-rest ratios of 20 s sprints to 40 s recovery. The HIIT was designed to cause a significant increase in T_c_ and decrease in blood pH. *Ad libitum* water ingestion was allowed only within the first 15 min of the trial to minimize interference with the T_c_ capsule readings during the trial. Five blood samples were taken over the trial (t = 45, 65, 85, 95, and 105 min post-caffeine ingestion) corresponding with pre-sauna (BASE), post-sauna (HEAT), post-steady state cycling (SS), post-HIIT cycling (HIIT), and 10 min into recovery after HIIT (REC), respectively.

### 2.5 Rest trial

The resting trial consisted of 105 min of inactivity (i.e., computer work, reading, etc.) and mimicked the exercise trial in caffeine dose, time of day, blood sampling timeline, and water limitation. Participants remained in the lab during this time.

### 2.6 Blood processing

Two samples of blood were taken at each timepoint. A small fraction of blood (∼1 mL) was collected in a heparinized vacutainer (BD Diagnostics, Franklin Lakes, United States) that was injected into an iSTAT CG4+ cartridge that was inserted into an iSTAT blood analyser (Abbott, Princeton, United States) for determination of blood lactate and pH. Another sample was collected in a 6 mL K_2_EDTA blood collection tube (BD Diagnostics, Franklin Lakes, United States) and the temperature was immediately recorded using a Long-stem Traceable® ULTRA™ thermometer (VWR International, Mississauga, Canada) with a precision of ±0.2°C. Hemoglobin was determined via Hemocue Hb 201 cuvettes (Hemocue AB, ÄNGELHOLM, Sweden), and hematocrit was measured in duplicate through microhematocrit tube centrifugation. The remaining blood was then centrifuged for 3 min using a PlasmaPREP (Separation Technology Inc., Sanford, Florida, United States) and designated as the total plasma concentration (*C*_*t*_). From this, a 1.0 mL aliquot of plasma was carefully transferred into the sample reservoir of a Centrifree® ultrafiltration device (Merck KGaA, Darmstadt, Germany), which was then spun in a fixed-angle rotor (Hettich fixed-angle rotor and Hettich Universal 320R centrifuge, Föhrenstr, Germany) for 10 min at a temperature consistent with the blood temperature recorded at collection to avoid the influence of ambient temperature on caffeine binding. This resulted in separation of *C*_*t*_ into the bound concentration (*C*_*b*_), which remained in the protein-rich plasma residing in the sample reservoir, and unbound concentration (*C*_*u*_), which was filtered along with protein-free plasma in the filtrate cup.

### 2.7 Plasma processing and analysis

Caffeine and paraxanthine were purchased from Sigma-Aldrich (St. Louis, United States) and caffeine-d9 and paraxanthine-d3 (Internal standards) were obtained from Toronto Research Chemicals (Toronto, Canada). All LC-MS/MS grade solvents were purchased from Caledon Laboratories Ltd. (Georgetown, Canada). Auto sampler vials/glass inserts used in the sample extraction were purchased from Chromatographic Specialties Ltd. (Brockville, Canada).

All samples were stored at −80°C until analysis. After allowing samples to thaw to room temperature, 100 µL (*C_u_*) or 200 µL (*C*_*t*_ and *C*_*b*_) were mixed with 10 µL of methanol and 10 µL of methanol containing 1 ng of internal standards (caffeine-d9 and paraxanthine-d3), followed by vortexing. Standard curves of caffeine and paraxanthine (10 points from 0.01 ng to 50 ng, 10 µL for each point of the curve) were prepared in the same conditions. Then, 1.2 mL of acetonitrile was added to each assay (standards and samples). After vortexing for 5 min, standards and samples were centrifuged at 20,000 g for 15 min at 4°C and the supernatants were transferred into 15 mL siliconized glass tubes before the addition of 3 mL of acetonitrile and being dried under nitrogen gas at 35°C. Samples were reconstituted in 120 µL of methanol, vortexed, centrifuged at 20,000 g for 20 min at 4°C, transferred to inserts and placed in the autosampler set at 4°C.

Samples were then injected (5 µL) and analytes were separated via high-performance liquid chromatography (HPLC; Agilent 1290, Agilent, Santa Clara, United States) using a Kinetex XB-C18 100 A (50 × 3.0 mm, 2.6 µm particle size) column (Phenomenex, Torrance, United States) with mobile phases consisting of 0.1% formic acid in water (A) and 0.1% formic acid in acetonitrile (B) at a flow rate of 0.7 mL/min, isocratic (15% B). Each run was 2 min.

The HPLC was coupled to a SCIEX QTRAP 5500 triple-quadruple mass spectrometer (SCIEX, Framingham, United States) equipped with a Turbo Ion Spray source. Electrospray ionization (ESI) was performed in the positive mode with multiple reaction monitoring (MRM) to select both parent and characteristic daughter ions specific to each analyte simultaneously from a single injection. The transitions used to quantify cbaseline were calculated using changes iaffeine and paraxanthine were as follows: caffeine (195.1 → 138.1), caffeine-d9 (204.1 → 144.1), paraxanthine (181.1 → 124.0), and paraxanthine-d3 (184.1 → 71.9). Nitrogen was used as the nebulizing, turbo spray, and curtain gas. Each target is then uniquely identified by the parent-to-daughter ion mass transition and the specific retention time. Data was collected and analyzed using Analyst v1.6.2 (SCIEX, Framingham, United States).

### 2.8 Plasma volume changes

Plasma volume changes (ΔPV) relative to baseline were calculated using changes in [Hb] and Hct, based on the equation by Dill and Costill (1974). Corrections were applied for the F-cell (whole blood vs. sample site) for Hct (x 0.91) and Hb (x 0.92), as well as for trapped plasma in Hct (x 0.96), as described by Novosadova (1977).

### 2.9 Determination of unbound fraction, metabolic ratio, and plasma volume changes

The %*f*_*u*_ of caffeine was calculated by dividing the concentration in the ultrafiltrate (*C*_*u*_) by the concentration in plasma *(C*_*t*_) and converted to a percentage (Equation 1). Additionally, the metabolic ratio (MR)—an indicator of the enzyme-mediated metabolism of caffeine and the associated production of its primary metabolite, paraxanthine—was determined for CYP1A2 by taking the quotient of *C*_*t*_ of [paraxanthine]/[caffeine] at each time point.

$$Unbound\;fraction\;\left(fu\right)=\frac{Unbound\;concentration\;\left(Cu\right)}{Total\;plasma\;concentration\;\left(Ct\right)}\times 100\%$$ (1)

### 2.10 Statistical analyses

Statistical analyses were performed using Prism (version 8, GraphPad Software Inc., San Diego, United States; RRID:SCR_002798) and an alpha level of .05. Plasma volume changes and drug concentrations were compared using 2-way, repeated measures ANOVA with treatment (Rest and EXS) and blood sampling timepoint (baseline, post-sauna, post-steady state cycling, post-HIIT, and 10 min post-HIIT) as within-subjects variables. In the case of missing values, we analyzed the data by fitting a mixed model as implemented in GraphPad Prism 8. Post-hoc analyses were applied to significant F-ratios yielded by the ANOVA using Dunnett’s multiple comparisons test to compare within-trial PV changes relative to baseline values, and Šidák’s multiple comparison test comparing isochronous values between Rest and EXS for all other measurements.

## 3 Results

### 3.1 Participants

The study was completed by all 10 participants that were recruited (Table 1). There were minor adverse events (i.e., nausea and vomiting) that occurred after the HIIT bout, but these resulted in no withdrawals of study participation. Of the 80 core temperature measurements, five displayed non-physiological readings (i.e., unexpected decreases in T_c_) and two were missing due to connection issues, resulting in their omission.

**TABLE 1:** Participant physical characteristics, aerobic power, and exercise intensities for EXS trial (n = 10).

	Mean ± SD
Age (y)	21.9 ± 2.7
Height (cm)	169.7 ± 6.4
Weight (kg)	67.4 ± 9.8
$\dot{V}O_{2\text{peak}}$ (mL⋅kg^−1^⋅min^−1^)	39.5 ± 4.4
Power output at 55% $\dot{V}O_{2\text{peak}}$ (W)	78.7 ± 20.4
Power output at 90% $\dot{V}O_{2\text{peak}}$ (W)	171.8 ± 36.6

SD, standard deviation; $\dot{V}O_{2\text{peak}}$ , peak oxygen consumption.

### 3.2 Plasma volume

**FIGURE 2:**
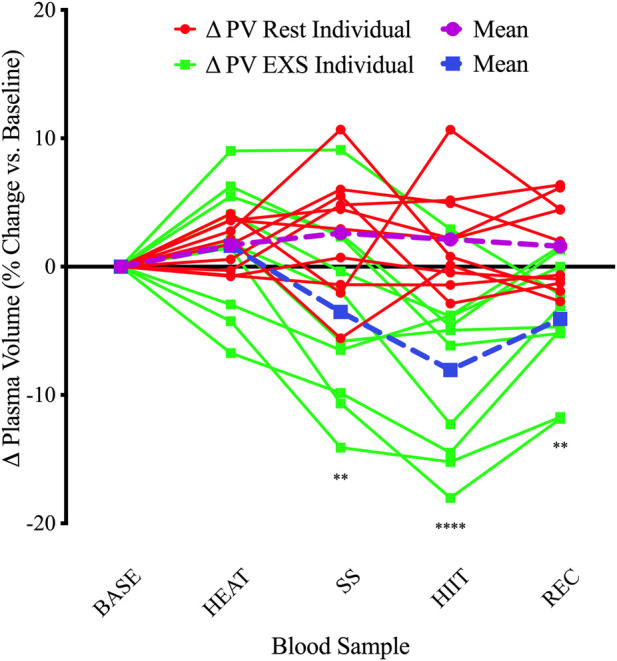
Individual changes in plasma volume compared to baseline. ΔPV, change in plasma volume for Rest and EXS trials; BASE, Baseline; HEAT, after passive heating; HIIT, after high-intensity intervals; REC, 10 min into recovery after exercise; SS, after steady-state cycling. **p < .01; ****p < .0001 significant difference compared to baseline.

Changes in plasma volume relative to baseline were determined using changes in [Hb] and Hct. In general, the Rest trial resulted in hemodilution while the EXS trial resulted in hemoconcentration (Figure 2). There were significant Time and Trial differences, and a Time × Trial interaction [F(4, 36) = 7.58, *p* < .0005]. Comparing mean changes to baseline using Dunnett’s multiple comparisons test, it was revealed that only after HIIT and into REC in the EXS trial were there significant differences (*p* < .0001 and *p* < .05, respectively). Comparing time-paired blood samples between trials with Šidák’s multiple comparisons test, significant differences were apparent after exercise was initiated (SS) and remained over the remainder of the trial.

### 3.3 Core temperature

**FIGURE 3:**
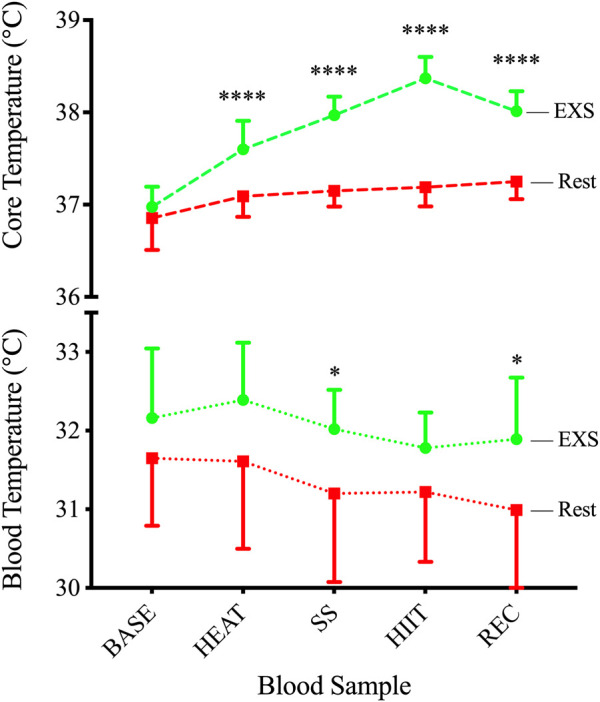
Core (TOP) and blood (BOTTOM) temperatures during Rest and EXS trials. *p < .05; ****p < .0001 significant difference between isochronous Rest and EXS values.

All participants completed 10 min of passive heating in the sauna, increasing T_c_ by 0.63°C above baseline before continuing to rise throughout the protocol and peak at 38.37°C ± 0.23°C during HIIT. This resulted in a significant Time × Trial interaction [F (4,29) = 34.61, *p* < .0001] via mixed-effects model, with T_c_ being significantly elevated over resting conditions (Figure 3, Top) at each time point after BASE. This increase in T_c_ was reflected in measurements of increased blood temperatures only after SS (+1.02°C; *p* = .040) and REC (+.90°C; *p* = .020) when measured immediately after sampling (Figure 3, Bottom).

### 3.4 Blood lactate and pH

**FIGURE 4:**
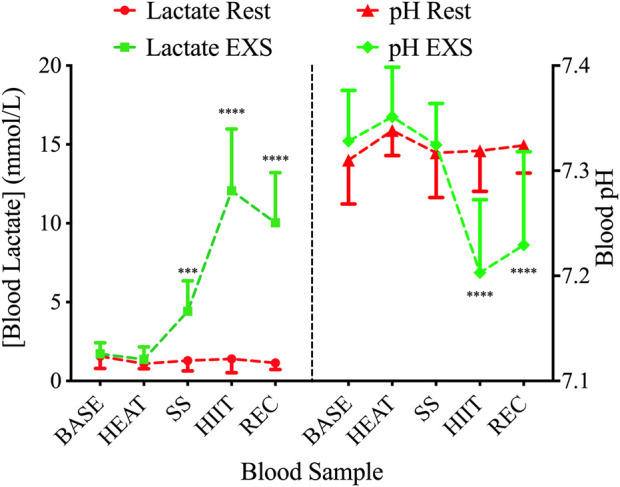
Blood lactate (left) and pH (right) during Rest (red lines) and EXS (green lines) trials. ***p < .001; ****p < .0001 significant difference between isochronous Rest and EXS values.

During the EXS trial, there was a significant Time × Trial interaction involving increases in blood [La^+^] after the commencement of exercise (i.e., SS, HIIT, and REC; F (4,36) = 72.88; *p* < .0001; Figure 4, Left). These increases were associated with a significant Time × Trial interaction for blood pH [F (4,36) = 12.71; *p* < .0001]; however, it was only after the high intensity exercise and into the recovery period (i.e., HIIT and REC) that *post hoc* analysis revealed a decrease during EXS (Figure 4, Right).

### 3.5 Plasma-drug concentrations

**FIGURE 5:**
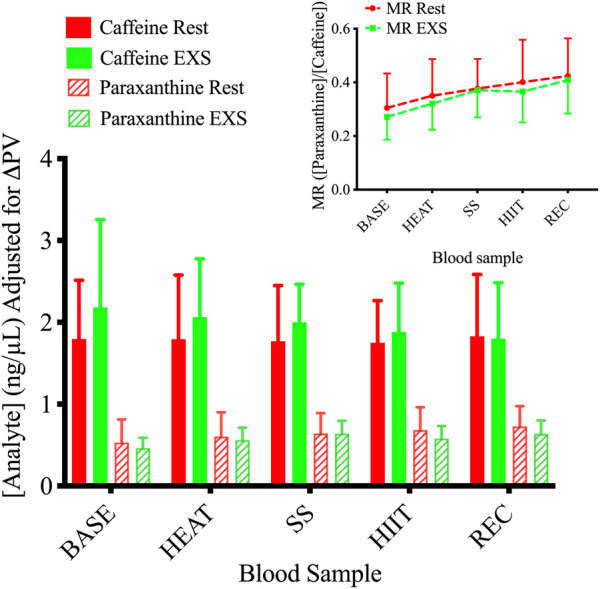
Plasma concentrations of caffeine and paraxanthine during Rest and EXS trials. Subfigure. Metabolic ratio (MR) of CYP1A2 measured as the ratio of [paraxanthine]/[caffeine].

ANOVA tests showed a Time × Trial interaction in paraxanthine plasma concentrations [F (4,36) = 2.662; *p* = .0482] but not of caffeine [F (4,36) = 1.038; *p* = .401] between Rest and EXS. Šidák’s post-test revealed no difference in paraxanthine values across time points between Rest and EXS. When blood concentrations were corrected for the relative changes in PV (Figure 5), the ANOVA interaction for paraxanthine was no longer significant. The MR of CYP1A2 (Figure 5, Subfigure) displayed only a Time effect [F (4, 36) = 15.89; *p* < .0001] as caffeine was metabolized to paraxanthine equally over the course of both trials. Post-hoc analysis was not performed since a Time × Trial interaction was not significant.

**FIGURE 6:**
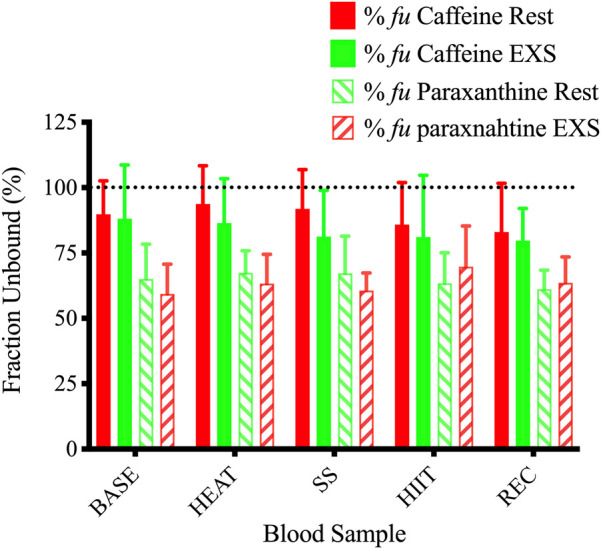
Unbound fractions of caffeine and paraxanthine during Rest and EXS trials. Dotted line shows 100% unbound fraction cut-off.

The %*f*_*u*_ of caffeine and paraxanthine did not differ across time points between Rest and EXS trials (Figure 6). The methods we used resulted in 23 of the 100 sampling points having %*f*_*u*_ values that were above 100%, which is impossible. Regardless, nearly all values were included in the analysis to allow for comparison between Rest and EXS. The values that were omitted were from a single participant that had a %*f*_*u*_ of >200% during their EXS trial as a result of relatively low caffeine *C*_*t*_ values. These samples were rerun but the new results were even more aberrant; therefore, these values were deemed unreliable, and all of their caffeine values (*C*_*t*_ and *C*_*u*_) were excluded.

## 4 Discussion

The *f*_*u*_ of a drug determines many PK and PD properties that can dictate its efficacy and potential for side effects. This is the first study, to our knowledge, to investigate *in vivo* changes in blood temperatures and pH on the *f*_*u*_ of a drug using ultrafiltration. An experimental protocol was designed with the intent of eliciting changes in T_c_ and blood pH, which are normal physiological responses to acute exercise. The results demonstrate that the intervention protocol was successful in raising T_c_ and decreasing blood pH compared to a non-exercise, control trial. However, the hypothesis that the intensity and duration of exercise used in the current study would lead to changes in T_c_ and blood pH that would affect *f*_*u*_ was not supported.

We used a combination of passive heating and various intensities of cycling to induce physiological changes in T_c_ and blood pH. T_c_ showed a gradual increase over the course of the interventions in the exercise trial with the greatest relative increase occurring from passive heating and the highest absolute temperature peaking at 38.37°C during HIIT. Even with this profound 1.37°C increase in T_c_ relative to BASE, and the swiftness of our research personnel to record blood temperature after collection in <1 min, blood temperature changed in the opposite direction to T_c_ but was still statistically slightly higher than time-matched results during the Rest trial after SS (+0.82°C) and REC (+0.90°C). Steady-state cycling was successful in maintaining a consistent blood pH while increasing T_c_, whereas the HIIT protocol effectively decreased blood pH while further increasing T_c_ compared to the Rest trial. Despite these pronounced changes, the plasma concentrations of caffeine and paraxanthine showed no difference between trials when corrected for PV fluctuations. Accordingly, the MR of CYP1A2 did not change during exercise, indicating that over the measurement period heat stress and exercise did not change the enzymatic rate of CYP1A2. Similarly, C_u_ did not differ between trials, resulting in no difference in the %*f*_*u*_ of caffeine or paraxanthine*.* Our resting data shows a %*f*_*u*_ of 89% (SD = 0.04%), which is at the upper end of the reported %*f*_*u*_ (70%–90%) (USIM, 2001), greater than the 64.55% reported using another ultrafiltration device (Blanchard, 1982b), and comparable to a study using the same ultrafiltration tubes (Lammers et al., 2018). Thus, our mean values appear acceptable; however, the same cannot be said for the variability, which might be masking potential differences. Of similar concern is that some of the %*f*_*u*_ values in our study were >100%, which is not physiologically possible. This, however, is consistent with other studies have also reported %*f*_*u*_ values above 100%. A major analysis of 25 epileptic medications showed mean unbound fractions of 102.7% for both gabapentin and pregabalin (Patsalos et al., 2017), which is slightly above their reported %*f*_*u*_ of 99% and 97%, respectively. This is obviously a smaller margin of error than the current study, yet still highlights the variability that is possible when deriving a value (e.g., %*f*_*u*_) using multiple mass spectrometry data. Nonetheless, there could be other factors that should be considered. For example, the use of protein precipitation in the sample preparation could be interfering with the caffeine concentration in C_t_. Although we did not validate the currently used assay under the full measures of the FDA guidelines (FDA, 2018), a protein precipitation method very similar to the one used in the current investigation has been validated with great % recoveries of caffeine and its metabolites recently (Chen et al., 2017). It is possible that the slight difference in reagents used (acetonitrile vs. formic acid (Chen et al., 2017)) resulted in differences in drug precipitating with protein, leading to the aberrantly low *C*_*t*_ values and resulting in %*f*_*u*_ >100%. Additionally, a long spin time might also artificially inflate the *C*_*u*_ as previously reported with valproic acid (Liu et al., 1992). This could result from perpetual flux from *C*_*b*_ to *C*_*u*_ according to the law of mass action as *C*_*u*_ is continually being spun through the filter and a true equilibrium is never attained. Findings by Kratzer et al. (2014) contradict this by showing no effect of centrifugation time on vancomycin, which they attribute to the constancy of *K*_*b*_, and that free protein and drug-bound protein are both concentrated in the sample reservoir at the same rate. Regardless, since all of the samples were processed similarly, a systemic effect on all the samples would have been apparent, which is not the case. This study highlights the need for standardized ultrafiltration methodology.

The cumulative thermal load (+1.37°C over BASE) during EXS in the current study is higher than the +0.9°C increase (36.9 → 37.8°C) reported previously by Kelly and colleagues (Kelly et al., 2016), who employed a similar protocol that had a 3 min warm-up at 60% $\dot{V}O_{2\max}$ , followed by fifteen high-intensity intervals (work:rest) of 30:30 s sprints at 90%:30% $\dot{V}O_{2\max}$ , respectively. Comparing the increases from exercise alone, however, reveals that their protocol elicited a slightly more elevated response in T_c_ (+0.9 vs. +0.8°C), which is likely a combination of their longer duration (7.5 vs. 3.3 min) of high intensity (i.e., 90% $\dot{V}O_{2\max}$ ) cycling and the higher T_c_ starting point of our participants who had just undergone passive heating (HEAT). Further comparisons show that our values are more in line with rectal temperatures reported by Reeve and colleagues (Reeve et al., 2019) (38.17°C), who performed a similar protocol of 10 min of passive heating, followed by a 6 min warm-up at 50% of work max (W_max_; watts), and then 12 × 1-min intervals at 100% W_max_ with rests of 1 min. Blood temperature may not mirror this increase because of differences in the measurement site. In our study, T_c_ was measured by a capsule situated in the GI tract. The temperature gradient has been reported to reach as high as 0.4°C between muscle tissues generating metabolic heat during exercise and the GI tract (Parkin et al., 1999). Therefore, blood flowing through arterial and venous conduits during exercise experiences temperatures ranging from ∼39°C at the muscle (Parkin et al., 1999) to ∼32°C in a superficial vein, based on our values. This reduction in temperature in mixed venous blood sampled from an arm vein is the result of combined efforts of radiative, convective, and evaporative heat loss, and is a display of our profound capacity to dissipate heat. This was elegantly displayed in exercising horses where temperatures of the gluteal muscle, pulmonary artery (representing T_c_), and superficial thoracic vein were 43.9, 42.8, and 39.5°C, respectively, after 10 min of running at 90% $\dot{V}O_{2\max}$ (Hodgson et al., 1985). This showcases the cascade of heat transfer and the rate at which blood is cooled in the vasculature during exercise. The greater difference between T_c_ and blood temperature in our study (6.6°C vs. 3.3°C) is likely a result of more efficient cooling in humans through a greater surface area to body weight ratio (Hodgson et al., 1985), better convection through our well-exposed dermis, the delay in measurement time after collecting the blood, and an evaporative response that begun in the sauna and was effective before the start of any exercise in our study.

The maintenance of blood pH over SS cycling can be explained by the increase in mean blood lactate ([La^−^]_b_) to 4.42 (range = 0.97–7.79) mmol/L, indicating that participants were very close to their lactate threshold, which is typically ∼4 mmol/L, yet varies inversely with training status (Stegmann et al., 1981). This increase in [La^−^]_b_ is greater than we anticipated since we had participants cycle at 55% of their $\dot{V}O_{2\text{peak}}$ with the intent to keep intensity below anaerobic threshold during SS cycling, which was previously reported to be 61.6% $\dot{V}O_{2\max}$ in untrained participants (Simon et al., 1985). Therefore, although lactate threshold was not determined in the present study, it is likely that SS cycling was performed near the individual lactate anaerobic threshold in the majority of participants. To efficiently lower blood pH, we chose a HIIT protocol with a work:rest ratio derived from work by Nicolo et al. (2014), which showed that “iso-effort” cycling at 20:40 s provided the largest increase in [La^−^]_b_ compared to 30:30 s, 40:20 s, and continuous cycling. In comparison, our participants engaged in 1/3rd of the HIIT volume in sets, yet displayed considerably higher [La^−^]_b_ levels (12.1 vs. 9.5 mmol/L), which is likely from a generally lower level of fitness (i.e., $\dot{V}O_{2\text{peak}}$ ; 40 vs. 67 mL⋅kg^−1^⋅min^−1^).

The calculated changes in PV were similar until initiation of cycling (i.e., SS, HIIT, and REC) in the EXS trial. Indeed, the %PV change after HEAT was comparable to the time-equivalent at rest (EXS: 1.63 ± 4.97% vs. Rest: 1.68 ± 1.87%); however, the variability was much greater in response to passive heating (Figure 2). This suggests that 10 min of passive heating may not be long enough to facilitate the same ∼5% hemodilution expected after 30 min of heating (Fortney and Miescher, 1994), which results from absorption of interstitial fluid into the vasculature in response to peripheral vasodilation and as an early compensatory mechanism for the ensuing sweating response (Bass and Henschel, 1956). This response shifts to hemoconcentration with the addition of exercise as the greater metabolic demands, coupled with an increased systolic arterial pressure, leads to greater vasodilation and an increased hydrostatic pressure of more capillary beds causing filtration to the extravascular compartments (Harrison, 1986). Additionally, since participants were instructed to abstain from drinking fluids after 15 min from the trial initiation, fluids lost from sweating during HEAT were not replaced and likely magnified the hemoconcentrated state during EXS since hydration status is a determinant of the direction of PV changes during exercise (Sawka et al., 1984). The 3.52% decrease in PV after 20 min of SS cycling at 55% $\dot{V}O_{2\text{peak}}$ is almost half of the previously described 6.8% decrease after 15 min of cycling at 67% $\dot{V}O_{2\max}$ (Novosadova, 1977). The 8.05% decrease at the end of HIIT is also shy of the reported ∼19% reduction after various HIIT protocols employed by Bloomer and Farney (Bloomer and Farney, 2013). After our participants stopped cycling, there was a classic PV rebound where they netted a +3.98% PV increase 10 min into REC. This is expected as exercise-induced decreases in PV tend to rebound to pre-exercise levels after a brief recovery period of ≥30 min (Novosadova, 1977).

Applying PV corrections avoids the inaccurate interpretation that total-body solute concentrations increase during hemoconcentration, and typically decreases the magnitude of change elicited by exercise (Berthoin et al., 2002). This is reflected in the current study where the Time × Trial interaction for paraxanthine was no longer significant when PV corrections were applied since variability from PV changes during EXS were accounted for. The results suggesting that caffeine is not sequestered in the intravascular space as fluid is expelled to the extravascular compartments during exercise is supported by previous research showing that [caffeine]_pl._ was no different after 60 min of walking at 30% $\dot{V}O_{2\max}$ (Collomp et al., 1991) and appeared slightly lower in lean individuals walking for 90 min at 40% $\dot{V}O_{2\max}$ (Kamimori et al., 1987). In proportion to the studies evaluating caffeine and exercise, relatively few researchers have compared the MR of caffeine and its metabolites during rest and exercise. One investigation in horses (Aramaki et al., 1995) revealed increases in the enzymatic conversion of caffeine to theophylline—the major metabolite in horses and converted by CYP1A2 and 2E1—in blood samples taken 3 to 71 h after 4,000 m of various intensity exercise. Theobromine, another metabolite, showed elevated conversion after 22 h of exercise, but paraxanthine, which is the only metabolite solely metabolized by CYP1A2, showed no change after exercise. Regardless, the combination of these processes resulted in caffeine’s half-life time (t_1/2_) to be hastened during exercise. This is congruent with post-exercise data in humans that showed a similarly faster t_1/2_ after 60 min of walking at 30% $\dot{V}O_{2\max}$ ; yet, MR changes cannot be substantiated since paraxanthine was not measured (Collomp et al., 1991). Based on these studies, it is possible that a longer sampling period in the current study would have revealed a change in the MR in the hours following the trial completion, or, simply, that the activity of CYP2E1, and not CYP1A2, increases with heat stress.

Given the complexity and time-dependence of measurements in the current study, there were some limitations that, if replicated, should be accounted for. The sample size met the *a priori* sample size calculation described previously in the Methods, but was relatively small, increasing the risks associated with extrapolation of our results to a larger sample size. At the time of our sample size calculations, very limited data were available to enable an *a priori* determination of the statistical power associated with that sample size; we thus determined power *post factum*. The results of our investigation can hopefully be used by those who will plan future related research, including an *a priori* determination of statistical power. The temperature of the sauna was not controlled by our study personnel since we lacked access to the controls. Controlling the temperature would allow for consistent heating among participants. Dermal and muscle temperatures were not measured. These values could describe the range of temperatures experienced by various drug-protein complexes and provide insight into thermal unloading of blood. Storing the vacutainers at mean venous blood temperature (e.g., 32°C), instead of room temperature, and insulating it upon collection would have minimized heat loss and provided greater confidence in the temperature of the blood sampled. To keep sample processing time to a minimum, the volume of fractions before (C_t_) and after separation (C_b_ and C_u_) were not measured, which precludes the possibility of calculating % recovery of C_u_ through the ultrafiltration method. Lastly, the results are only applicable insofar as they relate to the exercise employed and cannot preclude the possibility of changes in *fu* from greater perturbations in T_c_ and pH from other forms of exercise.

This is the first report of an *in vivo* study of healthy humans in which the effects on a drug’s *f*_*u*_ were assessed after a challenge involving passive heat stress and acute exercise. Passive heating followed by steady state and high-intensity cycling on a cycle ergometer were responsible for increasing T_c_ and decreasing blood pH 45–105 min after ingestion of caffeine. Despite previous reports on the necessity to control temperature and pH when analyzing drugs *in vitro*, we find no reason to be concerned about the effects of acute whole-body heat stress and exercise on the %*f*_*u*_ of caffeine. The validity and reproducibility of the methodology used to derive the *f*_*u*_ should be further investigated.

## Data Availability

The raw data supporting the conclusions of this article will be made available by the authors, without undue reservation.

## Glossary

ΔPV: change in plasma volume
ANOVA: analysis of variance
AGP: α1-acid glycoprotein
BASE: baseline/pre-sauna
*C*_*b*_: bound drug concentration
CL: clearance
*C*_*t*_: total plasma drug concentration
*C*_*u*_: unbound drug concentration
CYP#X#: Cytochrome P450 isozyme
EXS: heat-and-exercise trial
*f*_*b*_: bound fraction
*f*_*u*_: free/unbound drug fraction
Hb: hemoglobin
Hct: hematocrit
HEAT: passive heating/sauna
HIIT: high intensity intervals
*K*_*b*_: apparent binding constant
La^−^: lactate
MR: metabolic ratio
N-B: normal-to-base
N-F: normal-to-fast
PD: pharmacodynamics
PK: pharmacokinetics
REC: recovery period
Rest: resting drug trial
SD: standard deviation
SS: steady state
t_1/2_: drug half-life
T_c_: core body temperature
T_max_: timing of maximum drug concentration
V_d_: volume of distribution
$\dot{V}O_{2\text{peak}}$: peak aerobic power
W_max_: maximal work
